# A new age in functional genomics using CRISPR/Cas9 in arrayed library screening

**DOI:** 10.3389/fgene.2015.00300

**Published:** 2015-09-24

**Authors:** Alexander Agrotis, Robin Ketteler

**Affiliations:** MRC Laboratory for Molecular Cell Biology, University College London, LondonUK

**Keywords:** CRISPR, Cas9/sgRNA, high-throughput screening, high-content imaging, knockdown, siRNA

## Abstract

CRISPR technology has rapidly changed the face of biological research, such that precise genome editing has now become routine for many labs within several years of its initial development. What makes CRISPR/Cas9 so revolutionary is the ability to target a protein (Cas9) to an exact genomic locus, through designing a specific short complementary nucleotide sequence, that together with a common scaffold sequence, constitute the guide RNA bridging the protein and the DNA. Wild-type Cas9 cleaves both DNA strands at its target sequence, but this protein can also be modified to exert many other functions. For instance, by attaching an activation domain to catalytically inactive Cas9 and targeting a promoter region, it is possible to stimulate the expression of a specific endogenous gene. In principle, any genomic region can be targeted, and recent efforts have successfully generated pooled guide RNA libraries for coding and regulatory regions of human, mouse and *Drosophila* genomes with high coverage, thus facilitating functional phenotypic screening. In this review, we will highlight recent developments in the area of CRISPR-based functional genomics and discuss potential future directions, with a special focus on mammalian cell systems and arrayed library screening.

## Introduction

Functional genomics is a powerful technique to identify gene function, in particular through assessing cellular phenotypes arising from genome-wide perturbations. A classical approach to achieve such perturbations has been to alter the copy number of a gene, mRNA or protein. Historically, two opposing yet complementary methods have been established for this purpose: gain-of-function, including cDNA expression cloning; and loss-of-function, including short interfering (si) RNA knockdown experiments (Yao et al., 2015). These have been particularly useful for high-throughput screening approaches. Both cDNA and siRNA libraries are available from several commercial vendors in arrayed format, thus providing a convenient way of studying gene function on a genome-wide scale. However, conventional screening methods have lacked a certain degree of control over expression levels. For instance, cDNA libraries are often expressed at a high level that may raise questions over the physiological relevance of observed phenotypes. Nonetheless, expression cloning has proved a very useful tool, enabling discoveries which can be validated, for instance in the identification of virus receptors and cell surface receptor signaling effectors (Seed, 1995). The main method for genome-wide loss-of-function screening is using short hairpin (sh) RNA or siRNA libraries in order to reduce mRNA transcript levels. This has been successfully utilized to study aspects of mitosis (Neumann et al., 2006), virus un-coating (Kilcher et al., 2014), autophagy (McKnight et al., 2012), and cancer signaling (Schlabach et al., 2008), to name just a few examples. However, the levels of mRNA knockdown achieved are variable and frequently incomplete and off-target effects can occur that lead to non-specific knockdown (Buehler et al., 2012). More recently developed techniques utilizing genome editing and functionalization technologies have pushed the boundaries of gain- or loss-of-function experiments. It is now possible to make much more precise changes to endogenous gene expression level and protein function.

Genome editing involves making permanent changes to a genome DNA sequence using targeted nucleases, whilst genome functionalization refers to any type of targeted perturbation to the genome (e.g., gene activation, chromatin remodeling) that does not cause a permanent change in the DNA sequence. In the past, both approaches have been successfully performed using engineered zinc finger nucleases and transcription activator-like effector-based nucleases (TALENs) to study individual gene function, and also meganucleases have been used exclusively for genome editing (Joung and Sander, 2013). However, it has been pointed out that due to the challenges in construct engineering for these systems, which rely on protein–DNA interactions for targeting, it is difficult to exploit their potential in large-scale screening approaches in which many different genes must be individually targeted (Reyon et al., 2012; Heintze et al., 2013).

The recent development of clustered regularly interspaced short palindromic repeat (CRISPR)/Cas9 for experimental purposes has dismantled the perception that genome editing technology is off-limits for screening in mammalian systems (Heintze et al., 2013). Since this system employs the basic principle of Watson-Crick base pairing for gene targeting, generation of libraries with whole-genome target coverage is relatively easy and cost-effective. For instance, simple protocols are available to synthesize pooled lentiviral libraries by *in silico* design of oligonucleotides, which can then be cloned, packaged and delivered to cells by viral transduction (Paddison et al., 2004; LeProust et al., 2010). Similarly, the generation of arrayed libraries can be achieved by following protocols originally developed for arrayed shRNA library production that have been in use for a number of years (Moffat et al., 2006). All in all, the stage is set for CRISPR to make an enormous impact on genomic screening and thus scientific discovery in the coming years, and recent demonstrations of this system have shown great promise (Shalem et al., 2015). However, a number of technical challenges must be addressed in order to maximize the benefit of this technology. In this review, we will discuss current applications of CRISPR in functional genomics and provide a perspective on future developments in this area.

## CRISPR/Cas9 Genome Editing

CRISPR was identified as part of an adaptive immune mechanism in bacteria to destroy foreign DNA from sources such as bacteriophages and plasmids (Barrangou et al., 2007). Type II CRISPR systems integrate short sequence stretches of foreign DNA between CRISPR elements in the bacterial host genome. Transcripts from these repeats are processed into CRISPR RNAs (crRNA) containing the stretch of sequence (spacer sequence) resembling a part of the target foreign DNA (protospacer sequence). The crRNA can associate via base-pairing with another RNA that acts as a scaffold, the trans-activating CRISPR RNA (tracrRNA), and this RNA complex can then associate with the Crispr ASsociated (Cas) nuclease (Jinek et al., 2012). In the target DNA, the protospacer sequence is adjacent to a short (2–5 bp) sequence known as the protospacer adjacent motif (PAM) – together these direct the crRNA/Cas complex to the exact location in the foreign DNA, with the requirement for the PAM additionally ensuring that the host CRISPR locus (which lacks a PAM) is not targeted (Sternberg et al., 2014). Cas9 catalyzes cleavage of both strands of DNA at the site three nucleotides upstream of the PAM, resulting in degradation of the foreign DNA. This system therefore provides a form of host memory of the invading pathogen DNA, enabling the same pathogenic sequence to be targeted recurrently. The pairing of the RNA component with Cas9 also allows multiple distinct sequences to be targeted individually by a common Cas9 protein.

**FIGURE 1:**
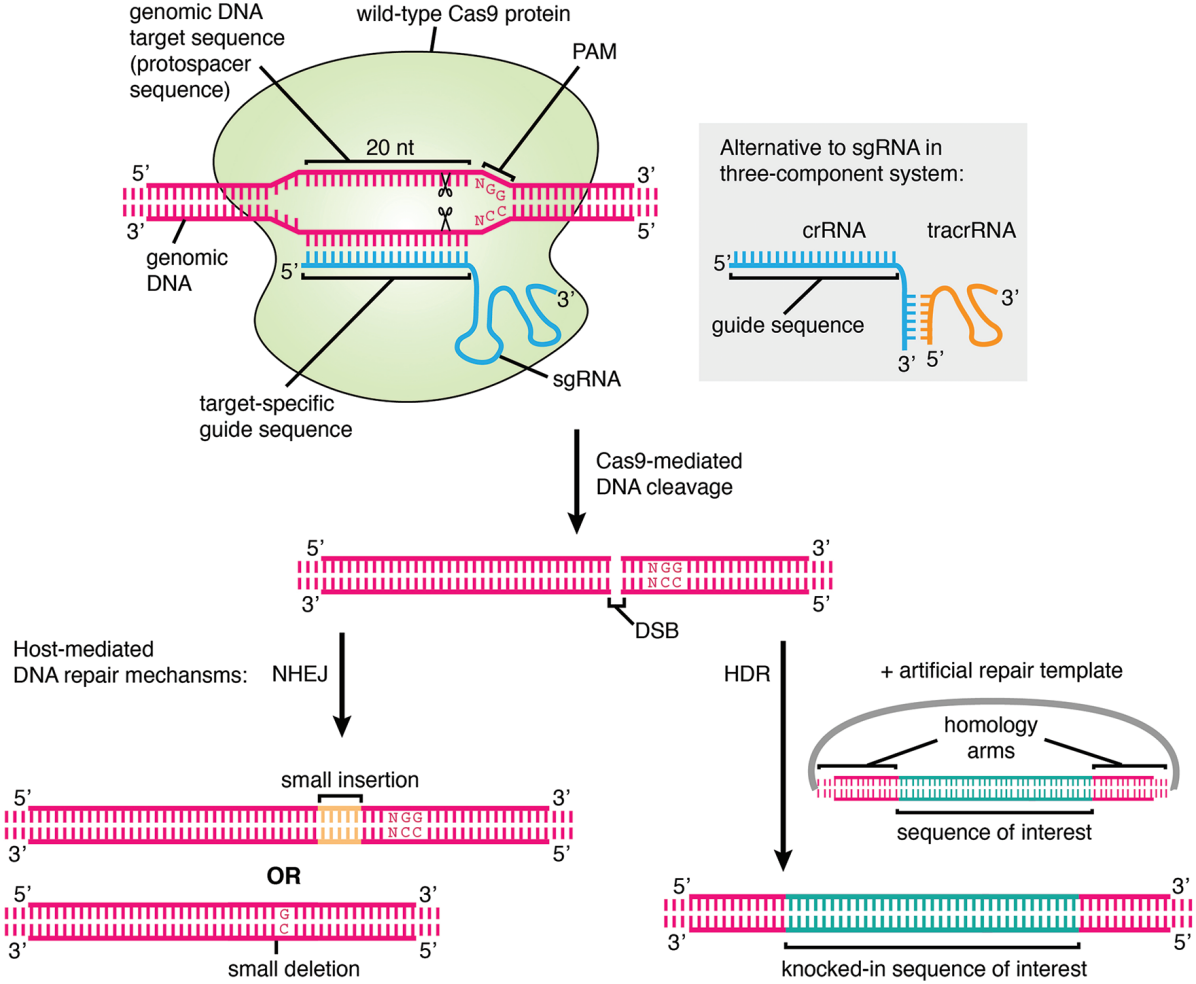
**The mechanism of genome editing using CRISPR/Cas9.** The genomic DNA target, which must lie adjacent to a protospacer adjacent motif (PAM), is specified by a 20 nt user-generated guide sequence in the sgRNA or crRNA. The *Streptococcus pyogenes* PAM is shown. In the cell nucleus, Cas9 protein associates with the sgRNA or crRNA/tracrRNA and binds to the target sequence, cleaving both strands of the DNA at the site 3 nt upstream of the PAM. Cleavage results in a DSB which is repaired by host-mediated DNA repair mechanisms. In the absence of a repair template, error-prone NHEJ occurs which may lead to the formation of random short indels and thus frameshift mutations and disruption of gene function, and this represents the main method of CRISPR-mediated gene knockout generation. If an artificial repair template is provided, for example on a plasmid containing a sequence of interest flanked by homology arms, then HDR may occur, leading to the introduction of an exogenous DNA sequence at a specified genomic location. This is the basis for performing gene knock-in, tagging, and precise pre-specified insertions or deletions using CRISPR. If catalytically inactive Cas9 is used instead of wild-type Cas9, then the protein simply binds to the target locus and does not cleave the DNA.

The type II CRISPR system from *Streptococcus pyogenes* has been adapted for targeted genome editing in eukaryotic cells (Cong et al., 2013; Jinek et al., 2013; Mali et al., 2013a,b). In a three-component system that is reminiscent of the natural system, the two RNA components (crRNA and tracrRNA) are introduced into cells or ectopically expressed along with a codon-optimized Cas9. When the crRNA and tracrRNA are artificially fused as a fully functional single “guide” (sg) RNA this becomes an even simpler two-component system, consisting of just the sgRNA and Cas9 (Jinek et al., 2013). The principle of targeting in both systems remains the same. The host genome of interest is targeted for disruption by identifying a suitable PAM motif and designing a targeting sequence specific for the adjacent region, to incorporate into the crRNA or sgRNA. This allows Cas9 to be recruited to the desired locus to exert its function, most commonly by forming double-strand breaks (DSB) in the DNA in the case of “wild-type” Cas9 (**Figure 1**). The presence of a DSB initiates host-mediated cellular repair pathways; in the absence of a repair template, non-homologous end-joining (NHEJ) is carried out. This error-prone mechanism frequently causes insertions or deletions (indels) in the DNA that can result in frame-shifting and disruption of the gene (Jinek et al., 2013). If a repair template is available then homology-directed repair (HDR) may occur. This principle is exploited for performing knock-ins using CRISPR; by providing an artificial repair template in addition to the two or three CRISPR components, one can efficiently introduce recombinant DNA into the genome at a defined location.

In addition to the targeting of Cas9 to a specific DNA sequence, the protein itself must be targeted to a specific cellular compartment, for instance by the attachment of a nuclear localization signal. Targeting other locations in the cell is possible in principle, and has been done for alternative genome editing methods such as TALENs, e.g., targeting to mitochondria by insertion of a mitochondrial targeting signal (Bacman et al., 2013).

Cas9 is presently the best-studied member of the Cas protein nucleases, although there are other members, and homologs in species other than *S. pyogenes* that have a similar role in bacterial host defense (Makarova et al., 2011; van Der Oost et al., 2014). Interestingly, type I and III Cas proteins require an additional component, the Cas3 helicase, to unwind the target DNA. This is not required for type II Cas proteins such as Cas9, explaining why this has been the preferred choice for use in genome editing technologies. As homologs of Cas9 vary in terms of the PAM sequence they recognize, different Cas9 proteins may be chosen to expand the range of genomic loci available for targeting (Shah et al., 2013), and recently *S. pyogenes* Cas9 was successfully engineered using a mutagenesis screen to obtain different PAM specificities (Kleinstiver et al., 2015). These developments are important because as the potential of CRISPR technology grows, so does the requirement for more precise targeting along with minimizing off-target effects.

One caveat of current CRISPR technology is that the Cas9 protein is rather large (~158 kDa in molecular mass), which has certain disadvantages, such as limiting viral titer when attempting to package the Cas9 coding sequence and consequently making this difficult to introduce into certain cell types. To help overcome this problem, a slightly smaller Cas9 from *Staphylococcus aureus* (approximately 124 kDa) has recently been described (Ran et al., 2015). Structural and biochemical studies have helped to reveal elements of the mechanism of Cas9 function and identify the domains involved in DNA binding; these may contribute to the design of smaller Cas9 proteins suitable for genome editing and functionalization. It has been noted from the crystal structure of Cas9 in complex with a guide RNA and target DNA (Nishimasu et al., 2014), and single-molecule based assays, that Cas9 itself contributes significantly to DNA binding (Jiang and Doudna, 2015). In fact, *in vitro* data suggests that PAM recognition by Cas9 precedes target site recognition by the RNA component (Sternberg et al., 2014). In addition, specifically targeted Cas9 remains strongly bound to its substrate DNA post-cleavage (Sternberg et al., 2014) and this feature may contribute significantly to the usefulness of engineered Cas9 in cells and *in vivo*.

## From Genome Editing to Genome Functionalization

The Cas9 protein has been engineered to obtain various properties that range from transcriptional repression to endogenous gene tagging (**Table 1**). In a more simplified view, Cas9 can be seen as the adaptor between the target sequence and a variety of functions. This reveals the most powerful concept of CRISPR technology: the ability to target a function to an exact genomic position. With this view in mind, it is conceivable to be able to design a minimal Cas9 protein with all extraneous regions deleted so that the protein simply binds the target DNA, and this would provide the most basic possible template for protein engineering. To date, successful CRISPR-based genome functionalization techniques have been based on fusing one or several functional domain to full-length catalytically inactive Cas9 (dCas9), which binds to the target locus but does not cleave the DNA. An important experimental consideration to take into account when following these approaches is that different sgRNAs must be designed for each functionalization in order to target the correct genomic features and achieve the desired output. For instance, transcriptional regulation requires sgRNAs that target promoter or regulatory regions, whereas sgRNAs used for knockouts most commonly target exons. Furthermore, the location of targeting within an individual gene can have a significant impact on the functional effect of the resulting mutation. For example, when using wild-type Cas9, targeting a coding region corresponding to a functional protein domain has been shown to be result in loss-of-function even for in-frame mutations, compared to exclusively targeting early exon regions, which often require frameshift mutations to achieve loss-of-function (Shi et al., 2015). Deliberately targeting certain gene regions can be used for achieving specific outcomes, such as knocking out a specific splice variant.

**Table 1:** Comparison of the different Cas9 functions that have been successfully demonstrated.

Function	Cas9 variant	Effector domain	sgRNA target	Detection method	Reference
Knockout and Knockin	WT nickase	– –	ORF	HRM; IDAA; Mismatch endonuclease;	Cong et al., 2013; Jinek et al., 2013; Mali et al., 2013a
	dCas9	Fok I		PAGE; Western blot; Sanger Sequencing;	Guilinger et al., 2014; Tsai et al., 2014
Activation	dCas9	Multimers of VP16 (VP48, VP64, VP160) p65 SunTag	Promoter	RT-PCR Western blot	Cheng et al., 2013; Gilbert et al., 2013; Maeder et al., 2013; Mali et al., 2013a; Tanenbaum et al., 2014; Konermann et al., 2015
Silencing	dCas9	KRAB	Promoter	RT-PCR Western blot	Larson et al., 2013; Qi et al., 2013; Gilbert et al., 2014
Gene tagging	dCas9	GFP/SunTag	Any	Immunostaining Microscopy Western blot	Tanenbaum et al., 2014
Genome locus visualization	dCas9	BFP EGFP GFP, 3xGFP, mCherry	Any	Microscopy	Chen et al., 2013; Ma et al., 2015
Optogenetic activation	dCas9	CIB1/CRY2	Promoter	Variable	Nihongaki et al., 2015; Polstein and Gersbach, 2015
Split reporter	Split Cas9	–	Any	Variable	Truong et al., 2015; Wright et al., 2015

*ORF, open reading frame; HRM, high-resolution melt analysis; IDAA, indel detection by amplicon analysis; PAGE, poly-acrylamide electrophoresis; VP16, tegument protein VP16 of herpes simplex virus; SunTag, peptide array that recruites multiple copies of an antibody fusion protein and GFP; KRAB, Kruppel associated box; EGFP, enhanced green fluorescent protein; BFP, blue fluorescent protein; CIB1, calcium and integrin binding 1; CRY2, cryptochrome circadian clock 2.*

Overall, the broad range of functional modalities coupled with an elegant targeting mechanism makes CRISPR systems very attractive compared to alternative functional genomics tools. For instance, cDNA libraries are restricted to gain of function applications and siRNA knockdown to mRNA silencing. Such simplifications can easily overlook the advantages of classical systems, however. For instance, siRNA applications do not require the introduction of a exogenous nuclease, and the long-term effect of Cas9 expression in cells and tissues has not been fully appreciated (Shalem et al., 2015). siRNAs are very effective in short time frames ranging from 1 to 3 days, making this an attractive approach for microwell based screening, since cultivation of cells within a microwell format for longer than 3 days poses challenges with overgrowth and subsequent high-content analysis of phenotype (**Table 2**).

**Table 2:** Key characteristics in CRISPR and siRNA technologies.

	siRNA	CRISPR
Targeting sequence	siRNA Oligonucleotide shRNA plasmid	(*Synthetic sgRNA*) sgRNA plasmid
Effector	- (Endogenous)	Cas9 Endonuclease
Effect	Knockdown (variable)	Knockout (Silencing, Activation)
Time to effect	2–3 days	Days; may require selection
Libraries	Lentiviral shRNA Pools Lentiviral shRNA arrays Arrayed siRNA Sub-panels	Lentiviral sgRNA Pools
Off-target effects	High	No consensus reached – Depending on policy of lab off-targets may be absent or very high

*For an overview of key differences between siRNA and CRISPR technologies, please see Taylor and Woodcock (2015).*

## CRISPR/Cas9 Screening

**FIGURE 2:**
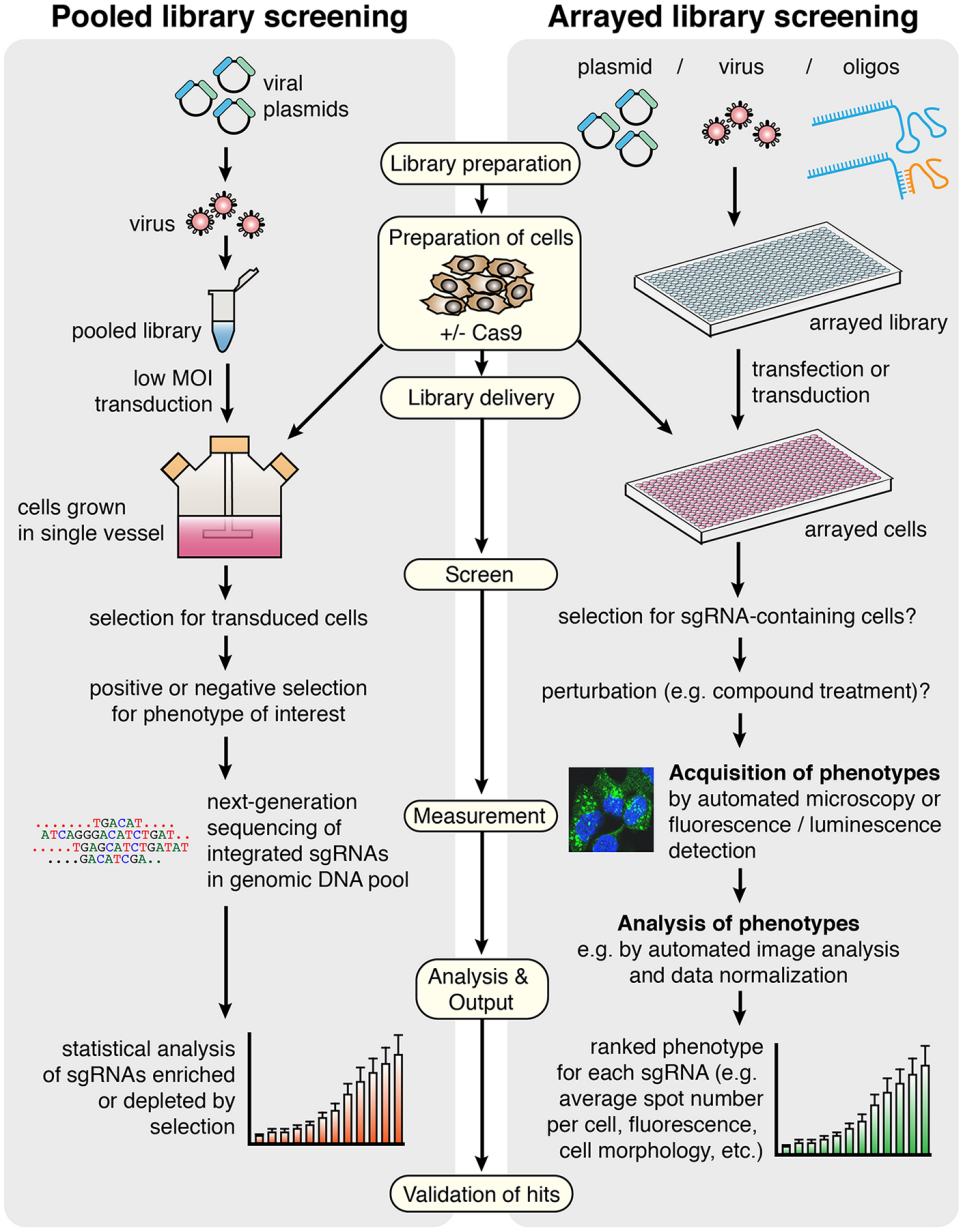
**General workflow for screening using CRISPR/Cas9 in pooled versus arrayed approaches.** In pooled screening, the viral sgRNA library is delivered to a single vessel of cells at low multiplicity of infection (MOI), before selections for transduced cells and specific phenotypes are carried out. The output of the screen is derived from deep sequencing of genomic DNA from the selected versus control cell populations, providing a measure of enrichment or depletion of each sgRNA in response to selection. In arrayed library screening, more library types and delivery methods are available since sgRNAs are delivered to discreet populations of cells grown in an arrayed format, preventing an individual cell from possessing multiple sgRNAs with different targets. There may be selection steps and treatments involved, but this can vary depending on the screen. Phenotypes are identified rather than necessarily being selected for, since the sgRNA responsible for each phenotype is known based on well location in the original annotated library. The final output for this method is a ranked phenotypic measure for each sgRNA delivered in the screen, and it may be chosen to detect multiple phenotypes in a single screen. In both methods, Cas9 can be stably expressed in the cells or co-delivered with the library. Key protocol steps that refer to both screening methods are indicated in pale yellow boxes.

A growing number of published studies have utilized CRISPR technology for screening (see **Table 3** for a comparison). CRISPR screening can be performed using pooled library approaches coupled to positive or negative selection, or alternatively arrayed libraries (**Figure 2**). To date, all studies have employed a pooled library approach, and whilst performed primarily as a proof-of-principle, these have uncovered novel genes that enhance or suppress simple phenotypes such as toxicity-induced cell death (Gilbert et al., 2014; Koike-Yusa et al., 2014; Shalem et al., 2014; Wang et al., 2014; Zhou et al., 2014; Konermann et al., 2015). They have also helped confirm important parameters of effective sgRNA design, which will undoubtedly assist the production of future libraries. A summary of these design considerations is listed in a recommended technical resource (Malina et al., 2014).

**Table 3:** Description of key published studies to date using pooled CRISPR-based screening libraries.

Study reference	Cas9 system	Library size, genes targeted	Cell type(s)	Selection	Key findings with implications for screening
Wang et al., 2014	WT Cas9 stably expressed.	73,151 sgRNAs 7,114 genes 10 sgRNA/gene	Human KBM7 (CML) HL60 (PML)	6-thioguanine Etoposide Proliferation	CRISPR-based KO screening is effective in both haploid and diploid cells. Off-target effects are minimal.
Shalem et al., 2014	WT Cas9 encoded in same lentivirus as sgRNA	64,751 sgRNAs 18,080 genes 3-4 sgRNA/gene	Human A375 (melanoma) HUES62 (stem cell line)	Proliferation Vemurafenib	CRISPR-based KO screening produces strong phenotypes that may be undetectable using RNAi. Hits have a high validation rate.
Koike-Yusa et al., 2014	WT Cas9 stably expressed	87,897 sgRNAs 19,150 genes 2-5 sgRNA/gene	Mouse JM8 (ES cell line)	Alpha toxin 6-thioguanine	CRISPR is powerful enough to be used for recessive screens. Off target effects are low, subject to effective sgRNA design. Lentiviral-delivered sgRNA guide sequences are most effective if the first nucleotide is a G.
Zhou et al., 2014	WT Cas9 stably expressed, OCT4 stably expressed to boost U6 promoter	869 sgRNA 291 genes ~3 sgRNA/gene	Human HeLa (adenocarcinoma)	Diptheria toxin Chimaeric anthrax	CRISPR has advantages over RNAi for knowledge-based screening with small focused libraries. It is important to select a clonal Cas9 cell line with a high modification efficiency.
Bassett et al., 2015	WT Cas9 encoded in same plasmid as sgRNA	40,279 sgRNA 13,501 genes ~3 sgRNA/gene	*Drosophila* S2R+ cells	Proliferation	First *Drosophila* whole genome sgRNA library. Plasmid transfection can be used in pooled approaches if DNA is diluted and cells are selected.
Gilbert et al., 2014	CRISPRi: dCas9-KRAB stably expressed CRISPRa: sunCas9 stably expressed	206,421 sgRNAs 15,977 genes ~10 sgRNA/gene 198,810 sgRNAs 15,977 genes ~10 sgRNA/gene	Human K562 (CML)	Proliferation Chimaeric cholera toxin-diptheria toxin catalytic A subunit	Genome-wide activation and repression-based screens are robust and give complementary results. Off-target effects are extremely low due to mismatch intolerance. Perturbation of non-coding elements is achievable.
Konermann et al., 2015	SAM (synergistic activation mediator) with protein components stably expressed	70,290 sgRNAs 23,430 genes 3 sgRNA/gene	Human A375 (melanoma)	Vemurafenib	The crystal structure of Cas9 can inform effective engineering strategies for gene activation. Activation-based screening is an alternative to cDNA overexpression, and hits have a high validation rate.

*CML, chronic myeloid leukemia; CRISPRa, CRISPR activation; CRISPRi, CRISPR interference; PML, promyelocytic leukemia.*

The basis of pooled library screening using CRISPR is firstly the production of a complex mixture of thousands of unique sgRNA-containing vectors, before delivery of the entire library to a single vessel of cells by viral transduction at low multiplicity of infection (MOI). Low MOI is absolutely crucial to limit the probability that an individual cell clone possesses more than one sgRNA, which would interfere with the assignment of genotype to phenotype in a mixed cell population. The sgRNAs can be designed with the aid of bioinformatic computational tools that assist in the identification of appropriate target sequences adjacent to PAMs, and libraries can be designed either with whole-genome coverage or for specific gene sets of interest, with single or multiple sgRNA designs per gene. The PAM is a fixed determinant in library design, restricting the number of possible targeting sequences in the genome. Nonetheless, it was estimated that every gene contains several such potential target sites (Montague et al., 2014), although this may differ for other genomic elements such as promoters. To date, no obvious targeting limitations imposed by chromatin accessibility or structure have been observed. The range of available sgRNA design tools is expanding and it can be beneficial to evaluate potential sgRNA sequences by taking advantage of multiple programs (Doench et al., 2014; Heigwer et al., 2014; Montague et al., 2014; Xie et al., 2014; Chari et al., 2015; Hodgkins et al., 2015; Naito et al., 2015; Prykhozhij et al., 2015).

In pooled screens, wild-type Cas9 or a modified derivative can either be stably expressed in the target cell line or encoded on the same viral vector as the sgRNA. After viral delivery of the library and selection for transduced cells, further selections are carried out for specific phenotypes of interest (for example by treatment with a compound which confers selective pressure for resistance phenotypes). The final step in the screen, which provides the read-out, is the deep sequencing of PCR-amplified genomic DNA from selected clones compared to an unselected control cell population, using sequencing primers targeting sgRNAs, to reveal the sgRNAs that are enriched or depleted following selection. Raw data of sequencing reads must be mapped against the original sgRNA library, annotated with the target gene that each sgRNA corresponds to, and statistical analysis is necessary to identify genes which are significantly likely to be relevant to the phenotype of interest. It is noteworthy that the sequencing step reveals the identity and relative abundance of integrated sgRNAs in the cell population rather than the occurrence of modifications (such as indels) at respective target loci, however the demonstrated high efficiency of CRISPR means that it is assumed that the majority of well-designed sgRNAs are successful in recruiting Cas9 function. Nonetheless, due to the incomplete knowledge of individual sgRNA efficiencies across a large library, and their varying potential for off-target effects, hits are typically only considered if multiple distinct sgRNAs targeting the same gene produce the same phenotype. The identification of known regulators provides assurance of a robust screen, and most prior studies have included additional internal controls to aid the evaluation of chosen experimental design.

Shalem et al. (2014) and Wang et al. (2014) and were amongst the first to demonstrate the feasibility of genome-wide loss-of-function-based screening using CRISPR and its advantages over siRNA in human cell lines. Whilst Wang et al. generated stable Cas9-expressing cell lines before transduction with sgRNA-containing lentiviral particles, Shalem et al. (2014) designed a “lentiCRISPR” vector containing adjacent sgRNA and Cas9 expression cassettes. Both systems made positive and negative selection screens possible. Positive selection, in which enriched sgRNAs are detected, has been adopted in all pooled studies and proved to be highly successful with few drawbacks. Negative selection, however, requires the screen to be carried out a large scale to ensure that entire library is represented multiple times over, providing sufficient sensitivity for depleted sgRNAs to be deduced from the final cell population. Zhou et al. (2014) proposed the benefits of smaller libraries focused on specific gene sets – an approach which is more broadly accessible and likely to be adopted for developing arrayed libraries. Bassett et al. (2015) generated a *Drosophila* whole genome sgRNA library where both sgRNA and wild-type humanized Cas9 are expressed from a single bicistronic expression plasmid. One caveat of this transfection-based system is that each cell will obtain multiple sgRNAs, resulting in lower signal levels. This can be overcome by dilution of the library in inert carrier DNA and will need to be tested in more detail.

Whilst the loss-of-function studies using wild-type Cas9 represent a milestone in demonstrating the utility of CRISPR for screening, we believe that CRISPR-based gene activation and repression approaches will prove very useful for functional genomics when further developed. The generation of gene knockouts with wild-type Cas9 has distinct caveats including: (1) the inability to study essential gene function, (2) the possibility that indels do not cause a frameshift and thus fail to affect gene function altogether, (3) the lack of phenotype in situations of redundancy or when cells do not express a particular gene, and (4) the reliance on host repair mechanisms to generate indels. It has been proposed that a combination of activation and repression-based screening is able to overcome these problems (Gilbert et al., 2014). CRISPR-mediated activation can successfully induce the expression of endogenous genes including those which are not normally expressed in the system of interest, as well as a full complement of protein isoforms, acting as a more exhaustive alternative to current cDNA expression screening. Gene repression using CRISPR can either utilize dCas9 alone targeted to promoters to block efficient transcription (Qi et al., 2013), or dCas9 fused to additional repressor complexes to greatly enhance the repressive effect. This has been demonstrated to be reversible, achieve greater knockdown efficiency than RNAi, and it can also be used to target non-coding RNAs (Gilbert et al., 2014). Limitations of these methods are nonetheless imposed by their complexity, since they require a larger Cas9 fusion protein and, in the case of activation, ectopic expression of synthetic transcription factor complexes. Konermann et al. (2015) designed a “Synergistic Activation Mediator” system consisting of dCas9 linked to VP64 (a fusion of four copies of the transcriptional activator VP16), in addition to a separate fusion protein of two distinct classes of activation domains (p65 and HSF1) linked to the bacteriophage coat protein MS2, which could be recruited to the target site via engineered loops in a modified sgRNA design. All of these components were required to achieve the most robust gene activation, which highlights the challenge of simplifying such systems in order to make them applicable for diverse screening platforms in different cell types.

## Arrayed CRISPR/Cas9 Screening

All pooled studies thus far have technically employed a forward genetics approach, whereby phenotypes are selected for and causative genetic modifications are revealed from downstream analysis. However, we envisage that the development of arrayed CRISPR libraries will enable reverse genetic screens with a much wider utility in terms of phenotypic read-out (including fluorescence/luminescence and image-based approaches); these may one day surpass the current use of siRNA-based approaches to become the default high-content screening (HCS) method. The technical challenges associated with the development of arrayed libraries, that have previously been a barrier for academic labs, are being overcome by industry and academic investment in CRISPR-based research tools. In fact, many academic labs and commercial companies are already engaged in the development of arrayed libraries.

Arrayed libraries are generated in multi-well plates, where each well contains a virus, vector, or reagent preparation targeting an individual gene or genomic locus. Using this type of library, it is possible to explore complex phenotypes (e.g., morphometric, subcellular localization of a fluorescent reporter) arising from a vast number of distinct cell perturbations in parallel, by delivering the library to cells grown in an arrayed format using automated equipment (**Figure 2**). DNA sequencing is not required in the initial screening workflow since cells exhibiting a phenotype of interest can simply be cross-referenced with the annotated library to reveal the delivered sgRNA.

Overall, the requirements for arrayed library screening are very different from approaches using pooled libraries. Whilst the experimental conditions will ultimately vary between screens, there are at least five important steps to be considered by the researcher in arrayed CRISPR screening (**Figure 2**):

(1) Preparation of cells;
(2) Library delivery;
(3) Acquisition of phenotypes;
(4) Analysis of phenotypes;
(5) Validation of hits.

### Preparation of Cells

Ideally, arrayed library screening is performed using cells grown in 96- or 384-well microwell plates. Compared to siRNA-based screening, arrayed library CRISPR-based screening may need to be performed on a longer timescale (**Table 2**). Typically, siRNA results in a detectable reduction of mRNA levels within 24–72 h, thus making the time from delivery of the siRNA to detection of a phenotype relatively short. In contrast, the kinetics of DSB induction and repair have not been fully characterized in the context of CRISPR, and they are likely to differ from the kinetics of natural DSB repair due to Cas9 remaining bound at the target following cleavage. Furthermore, following repair more time may be needed before a phenotype is observed, since this requires the gene transcript and protein level to deplete naturally, in contrast to using si/shRNA which targets transcripts directly. In pooled applications, at least one week is usually given to allow repair of the DSB followed by depletion of the transcript and protein, including selection for transduced cells using an antibiotic resistance marker or flow cytometry based sorting. Given these considerations, the initial number of cells seeded into plates in an arrayed screen may need to be rather low. For some genes, knockout or perturbation will significantly affect cell fitness, resulting in unequal cell growth across individual wells of a plate. This poses a challenge with downstream selection protocols and normalization of cell numbers across samples. It can also affect experimental data, since some HCS based phenotypes, such as cell polarity, utilize parameters that are affected by cell density. In addition, overgrowth of cells can result in artifacts due to stress. Large-scale screens require automated handling equipment that cannot routinely re-plate cells in a subset of wells from one plate to another. For these reasons, when using arrayed libraries it is recommended to optimize an effective screening workflow that minimizes the time frame between perturbation and measurement.

A major point of consideration is whether to use a stable Cas9-expressing cell line or to introduce Cas9 together with the sgRNAs. Stable Cas9 cell lines are much easier to handle and reduce inconsistencies that could arise from variable Cas9 expression or activity levels within a cell pool. However, clonally derived cell lines may exhibit phenotypic artifacts. It has also been suggested that co-delivery of Cas9 and sgRNA on a single vector ensures consistent stoichiometry of both components, which might be preferable to variable sgRNA expression in a consistent Cas9 expression background (Malina et al., 2013). Furthermore, many cell types such as primary cells do not lend themselves to stable line generation. The recent generation of Cas9 transgenic mice (Platt et al., 2014) not only enables *in vivo* CRISPR-based experiments; it also provides the valuable opportunity to isolate primary mouse cells for *ex vivo* culture that stably express Cas9 without the need for further manipulation, requiring only the delivery of sgRNAs for screening (Shalem et al., 2015). If such an approach proves effective, it will certainly be very useful to generate other mouse lines expressing tissue-specific or functionalized Cas9 variants in order to perform CRISPR activation and repression-based screens in primary cells. These systems can also be made inducible to allow for temporal control of Cas9-mediated activity or functions, as well as reducing any potential negative effects of long-term Cas9 expression.

An alternative to using stable Cas9 cells or transient transfection of Cas9 expression plasmids is the delivery of recombinant Cas9 protein. Cas9 protein is available from multiple commercial suppliers (NEB, Toolgene). This approach was first described for delivery of Cas9/sgRNA ribonucleoprotein complexes by microinjection into *C. elegans* (Cho et al., 2013) and was subsequently used to generate gene knockout mice and zebrafish (Sung et al., 2014). Both, electroporation and lipofection-based methods for delivery have been described (Kim et al., 2014; Zuris et al., 2015). In this case, it has been proposed that the time from delivery to analysis using *in vitro* transcribed sgRNA and Cas9 protein transfection can be less than 3 days, making this comparable to siRNA-based approaches. Nuclease-mediated indel rates with these methods were as high as 94% in Jurkat T cells and 87% in induced pluripotent stem cells (Liang et al., 2015). Generally, these methods achieve high efficiency of indel generation and low off-target effects. Recently, ribonucleoprotein complexes of Cas9 and sgRNA were also used with donor DNA for HDR in various cell types (Lin et al., 2014). Despite these promising reports, the current cost of recombinant Cas9 protein is likely to be prohibitive for its use in arrayed library screening.

### Library Delivery

The nature of arrayed library screening means that a range of library types and delivery methods are available. Library types can broadly be grouped into those based on plasmid or viral expression vectors, and those using synthetic oligonucleotides. Screening with CRISPR could use multiple possible configurations, which could include combinations of a stable cell line expressing Cas9 plus lentiviral-delivered sgRNAs, plasmid-based or synthetic guide RNA. Alternatively, transient expression of Cas9 or transfection of recombinant Cas9 protein can be used in combination with the guide RNA. It is also possible to generate screening libraries for any of the different functionalities. In **Table 1**, we have summarized the different requirements for sgRNA library design depending on the functionalized Cas9 that is being used.

A key advantage of vector-based systems is that bi-cistronic expression of a selection marker is possible, allowing for selection of Cas9- and/or sgRNA-expressing cells. This can be an antibiotic (e.g., puromycin) resistance marker, or a fluorescent protein. Both methods of selection work reasonably well and choice of selection method is mainly guided by the availability of the respective tools, and whether incompatible reporters are used for Cas9 expression selection or for phenotypic measurements. It may be best to avoid selecting cells using antibiotics that rely on the introduction of DNA double strand breaks such as zeocin or bleomycin. In arrayed screening coupled with high-content microscopy, fluorescent selection would likely be done through segmentation of fluorescent cells in image analysis rather than through flow sorting, which is better suited for pooled approaches.

In expression vectors, RNA polymerase III-dependent transcription of the sgRNA is driven by a U6 or, less commonly, a H1 promoter. A direct comparison of these two promoter types has not been done to date. Previous analysis of U6 and H1 promoter mediated transcription has shown that in certain cell types such as endothelial cells and in mouse brain, the U6 promoter is more efficient (Makinen et al., 2006). Whilst the U6 promoter has a requirement for the base “G” to initiate transcription, this does not necessarily limit potential target sites since it was shown that always using G as the 5′ nucleotide of an sgRNA is preferable for its expression, and remains efficient (at least for knockout-based screens) regardless of whether this matches the intended target sequence (Mali et al., 2013b; Koike-Yusa et al., 2014). H1 mediated transcription can result in variability of small RNA transcripts due to multiple start sites at the 5′-end (Ma et al., 2014), making U6 promoters the overall preferred choice for sgRNA expression. An alternative is the use of very short tRNA promoters to express cleavable tRNA–sgRNA chimeras (Mefferd et al., 2015), but a more detailed analysis of this strategy remains to be done.

#### Lentiviral Vectors

At present, the most common method for sgRNA library expression is through the use of lentiviral vectors. Additionally, most companies place an emphasis on generating lentiviral libraries, both pooled and arrayed, presumably due to the limited processing and library delivery steps required by the end user. However, handling such libraries requires access to biosafety level 2 facilities which can be very costly. The main advantage of using lentiviruses is the ability to infect a broad range of cell types with high efficiency, and without the need for additional delivery reagents. For arrayed screens, infection could be carried out at high MOI to ensure that the majority of cells express one or more copies of the sgRNA expression cassette.

Lentiviruses integrate into the host genome, maintaining sgRNA expression over a long period of time, which has both advantages and disadvantages for screening. On the one hand, sustained sgRNA expression may increase the likelihood of on-target modifications over time, and indeed, this has previously been seen to be the case and is particularly noticeable for less efficient sgRNAs (Shalem et al., 2014). On the other hand, it may increase the likelihood of off-target modifications. Also, integration events occur at random sites, potentially leading to artifacts such as oncogene activation when looking at clonal populations of cells. It has not been conclusively shown whether such long-term expression is an absolute requirement for CRISPR-based knockout screening, as transient delivery of sgRNA or crRNA works reasonably effectively (Kim et al., 2014; Glemzaite et al., 2015). However, for silencing or activation screens, it remains to be seen how rapidly dCas9 may dissociate from the target site if guide RNA expression is not maintained. If this proves to be an issue, then the long-term sgRNA expression afforded by lentiviruses will make them the preferred vector choice for such screens.

#### AAV Vectors

Increasingly, adeno-associated viral (AAV)-based vectors have been used for delivery of the sgRNA to cells, and have been particularly successful for *in vivo* delivery of Cas9 and sgRNAs to the brain (Swiech et al., 2015). AAV-based vectors are non-integrating, making it more difficult to identify sgRNA traces in pooled approaches by next-generation sequencing, although they can be detected by episomal sequencing. Since there is no requirement for sgRNA sequencing during arrayed screens, AAV vectors are well suited for this application, especially if integration and long-term sgRNA expression are a particular concern, but efficient delivery is required.

#### Plasmid Vectors

The classic approach for delivering sgRNAs in a standard CRISPR experiment is through transient transfection of a plasmid vector, and arrayed screening allows this delivery method to be adopted. Plasmids do not suffer from the same size constraints as viral vectors, therefore the convenient delivery of Cas9 and sgRNA expression cassettes on the same plasmid is common. Plasmids libraries have the advantage of being replenishable by propagation in bacteria. Some cell types, such as HEK293T cells, lend themselves well to plasmid-based screening, since they can be transfected with efficiencies close to 100% using standard transfection reagents such as calcium phosphate, polyethylenimine, and lipid-based reagents. However, a number of other cell types including primary cells are refractory to these transfection methods; electroporation is typically used to overcome such problems (Luft and Ketteler, 2015). It can be difficult to control the expression level of Cas9 and sgRNA by plasmid transfection, therefore this method may impose a trade-off between transfection efficiency and possible off-target activity and artifacts caused by overexpression of sgRNAs and Cas9.

#### Synthetic Oligonucleotides

An alternative to the use of vector-based systems is the delivery of synthetic oligonucleotides. In principle, it is possible to generate RNA only libraries of the sgRNAs. A transient transfection approach for sgRNAs would be carried out in a similar manner to siRNA screening, which is currently the most popular method for genome-wide functional studies. Whilst the length of an individual sgRNA in nucleotides is much longer than RNA in classical siRNA libraries (>100 nt compare to 21 nt), they can still be commercially synthesized with reasonable accuracy, albeit at a greater cost.

Co-transfection of the target-specific crRNA plus a common tracrRNA is feasible and has been successfully used (Kim et al., 2014; Glemzaite et al., 2015), bringing the possibility of generating a library of crRNAs to co-introduce with the tracrRNA. This arrangement is already used alongside transient Cas9 transfection in the Edit-R system from Dharmacon, with other companies, including Sigma-Aldrich and ThermoFisher, currently exploring similar options. Since the tracrRNA can be commercially obtained in large quantities, using a synthetic three-component system is generally the most cost-effective option for screening. The crRNA has a 20 nt sequence specific to the genomic DNA target site plus another 20–30 nt sequence for interaction with the tracrRNA, making it considerably shorter and cheaper to synthesize than an entire sgRNA. To date, a single delivery system based on the sgRNA has been favored for simplicity. Whilst relatively costly for large arrayed libraries, this may be a very reasonable option for limited sets of pathway genes, and is affordable compared to current pricing models for lentiviral sgRNA constructs. Furthermore, chemical modifications to the sgRNA can improve genome editing efficiency, in particular when co-delivered with Cas9 mRNA or protein (Hendel et al., 2015). It can be expected that such modifications will result in improved library design similar to advances in siRNA library design by chemical modifications that resulted in enhanced on-target and reduced off-target activity (Mohr et al., 2014).

Such platforms may allow phenotypic analysis within 3 days after delivery since they do not depend on integration and/or expression to have an effect, but the efficiency of this technology needs to be explored. This approach remains attractive compared to other delivery methods, both in terms of overall costs and reducing the number of steps necessary to prepare delivery reagents for screening, and thus we recommend that oligonucleotide-based CRISPR screening libraries and technology should be developed further in the future. One potential disadvantage, however, is the inability to easily select for cells that have taken up synthetic guide RNA. One possibility is to label the transfected RNA with fluorescent probes. Several companies offer kits for labeling siRNA with fluorescent dye to visualize transfected cells without affecting efficacy. It remains to be seen whether this is also possible for guide RNAs.

### Acquisition of Phenotypes

Pooled libraries require strong phenotypes that can be enriched by positive or negative selection (Shalem et al., 2015). This has limited the use for high-content imaging based methods that observe complex cellular phenotypes as opposed to simple binary responses. The availability of arrayed siRNA libraries in the early 2000’s greatly facilitated the use of HCS based methods in functional genomics (Kiger et al., 2003). A wide range of cell-based phenotypes can be visualized including morphological features (Kiger et al., 2003; Yin et al., 2013), organelle dynamics (Ferraro et al., 2014), protein trafficking (Liberali et al., 2014) and post-translational protein modifications such as protein phosphorylation (Papageorgiou et al., 2015). An overview on HCS and phenotypic analysis is given elsewhere (Liberali et al., 2015). The analysis of phenotypes in a typical HCS experiment can be complex and involve multivariate profiling of hundreds of features (Loo et al., 2007; Rameseder et al., 2015). Some HCS assays such as those based on protein translocation enable the identification of striking phenotypes in a small sub-set of cells (Freeman et al., 2012), but in most cases a high penetrance of the phenotype in the majority of cells is required. This has been achieved using siRNA based methods coupled to sophisticated image analysis programs so that even heterogeneity in cell populations can be determined (Snijder et al., 2012). The penetrance of a phenotype (i.e., the number of cells displaying a phenotype upon introduction of the perturbation) elicited by CRISPR/Cas9 has not yet been determined. Efficiencies of knockout using the CRISPR/Cas9 system have been reported in the range from 10 to 90 % and are dependent on the choice of cell line. Overall, it is not clear whether sgRNA or crRNA libraries will enable robust phenotype identification without the necessity of a selection step. Alternatively, it might be necessary to use co-delivery of reporter genes in order to tag and identify the guide RNA-containing cells, assuming that the actual modification efficiency is very high.

### Analysis of Phenotypes

High-content screening assays can involve multi-parametric analysis, although in reality most published high-content screens only use a limited number of features for analysis (Singh et al., 2014). For this purpose, most HCS platforms come with a commercial high-content analysis package such as InCell Analyzer (GE Healthcare), Harmony (PerkinElmer Inc), or Metamorph (Molecular Devices, LLC.). In addition, multiple software programs for high-content analysis exist that are freely available to the community, such as CellProfiler (Carpenter et al., 2006) and ImageJ/Fiji (Schindelin et al., 2012). Detailed protocols for high-content analysis are available elsewhere (Misselwitz et al., 2010; Stoter et al., 2013).

### Validation of Hits

Whilst it would be preferable to include validation steps a typical arrayed screening protocol to minimize bias, current validation methods are highly resource-intensive which limits the ability to carry them out on a large scale. For example, detection of indels requires specific primer sets that cover each target locus; these would need to be designed and tested, representing an enormous cost that could easily dwarf the cost of the screen itself. Therefore it is recommended to focus on validation of hits post-screen. The techniques available for doing this can also be usefully implemented for initial screen development, such as for comparing the efficiency of different guide RNA and Cas9 delivery methods when targeting a small set of test genes.

Common standards for validation of hits and rescue of phenotypes in CRISPR experiments have yet to be agreed. However, it is quite important to verify exact genome modifications at the sequence level to rule out artifacts. For instance, the introduction of indels can lead to functional consequences other than disruption of the gene, such as expression of alternative frameshift proteins (Ketteler, 2012). Different types of validation experiments will be required for screens utilizing different CRISPR methods. All methods targeting protein-coding genes benefit from analysis of protein expression level by western blot if primary antibodies are available, and RT-qPCR can be used compare transcript levels where this is not possible or when non-coding RNAs are targeted. In addition to detection of the underlying genome modification and its effect on transcription and translation, it is important to validate gene-specific effects (and thus rule out off-target effects) by rescue experiments or reproducing a phenotype using multiple independent guide RNA sequences, as discussed in more detail below.

#### Detection of Genome Modification

There are two categories of detection methods. The first is to detect whether a change has occurred in the DNA sequence; these methods are typically used for initial identification of modified cell pools and comparing overall modification efficiencies. The other is to detect the exact genome modification, for instance by Sanger or next-generation DNA sequencing for gene knockouts/knockins or methylome analysis for epigenetic changes, however, this approach requires clonal cell populations.

A quick method for identification of indels is based on enzyme mismatch cleavage assays (Langhans and Palladino, 2009). The surveyor nuclease Cel1 or T7 endonuclease T7E1 are mismatch-specific DNA endonucleases that will cleave mismatched heteroduplex DNA and produce two smaller fragments that can be resolved by gel electrophoresis. A recent comparison between Cel1 and T7E1 indicates that T7E1 enzymes preferentially cleave deletions, whereas Cel1 is able to detect single nucleotide changes (Vouillot et al., 2015). An alternative method uses native poly-acrylamide gel electrophoresis (PAGE) to identify indels (Zhu et al., 2014). In this method, PCR fragments of the target region are denatured and re-annealed resulting in homo- and hetero-duplexes. Heteroduplexes of DNA with a mismatch harbor an open angle between the matched and mismatched DNA strand, which will result in slower migration during native PAGE. Thus, even in the absence of a mismatch endonuclease, a different migration pattern can be detected that is indicative of indels. However, these assays often lack quantitative sensitivity and are relatively low-throughput.

A more scalable and sensitive method to identify indels in cell populations is to use high resolution melt (HRM) analysis (Price et al., 2007; Dahlem et al., 2012). In this method, amplicons of 50–300 bp are generated to allow detection of differences in the melting curves when indels are present (Thomas et al., 2014). HRM analysis can also be used to detect off-target mutations. This analysis can be done on standard qPCR machines that are present in most research institutes. One limitation is that deletions larger than the selected amplicon size will not be identified. A variant of this method uses automated capillary electrophoresis (Yang et al., 2015). In this method, the amplicon is labeled with fluorescent tags using tri-primer PCR. The fluorescently labeled amplicons are then detected by fragment analysis on an ABI3010 sequenator. This method can reliably identify as little as single nucleotide changes and kits for amplicon labeling are commercially available. The main disadvantage is that this method requires equipment for fragment analysis such as the ABI3010 that is a major added expense for most labs.

More complex systems include reporter cells to measure DSBs, such as the Traffic Light Reporter (Kuhar et al., 2014), which can reliably and simultaneously measure NHEJ and HDR. This requires the introduction of GFP/mCherry cassettes into cells and thus is not particularly practical for post-screening validation. However, this technology may have utility during initial screen development, as long as the reporter is introduced into the same type of cell that will be used for the actual screen, since different cell types are expected to have different modification efficiencies.

Detecting an exact genome modification in clonal cell populations can be achieved by next-generation sequencing or Sanger sequencing. Sanger sequencing is more suitable for haploid cells, since multiple allelic modifications would likely give rise to a mixed sequencing trace, making it difficult to interpret exact sequence changes. One limitation for Illumina or 454 sequencing is that due to the short read-length, the detection of large modifications cannot be accomplished. A sequencing-based method that produces larger amplicons termed single molecule real time sequencing (SMRT) can generate read lengths of up to 15 kb and is suitable for detecting large-scale genomic changes (Hendel et al., 2014).

#### Rescue of Phenotype

With a lack of standards for validation of CRISPR-generated phenotypes, problems arise from the analysis and relevance of clonal populations. A key question is whether one needs to consider multiple clones for confirmation of phenotypes, and if so, how many? It seems that generating clones using different guide sequences targeting the same gene provides the best way to validate a phenotype, but this may not always be possible due to limitations imposed by the PAM and the potentially variable functional outcomes of targeting different loci within a gene. Further, a desired feature for validation is to revert a phenotype by rescue experiments. For knockout or repression phenotypes, this can be achieved by cDNA expression. For activation phenotypes (e.g., by CRISPRa), this can be achieved by si/shRNA-mediated reduction of the gene transcripts. It will be important to revert expression levels back to physiological levels, but titrating the exact expression levels can be a challenge with classical cDNA and si/shRNA techniques. An alternative is to use BAC-mediated expression to achieve more physiological levels of gene expression (Poser et al., 2008), or to apply CRISPR-mediated genome engineering (e.g., gene knock-in), but the latter might be challenging in cases where indels largely disrupt genes. Inducible systems would be desirable, but these are not applicable to irreversible modifications such as the generation of knockouts. In general, this is an issue that requires further experimentation and discussion before a consensus is met between CRISPR users within the scientific community.

## Conclusion and Outlook

Overall, the use of CRISPR based methods in high-throughput functional genomics screening is still in its infancy. The first pooled libraries show encouraging results, but many technical considerations need to be explored for the development of arrayed libraries. The generation of large-scale libraries is possible not only for human and mouse, but virtually any organism. In the past, siRNA libraries have mostly focused on *Drosophila*, *C. elegans*, human, mouse, and rat genomes, though in principle has always been possible to design and produce libraries for other organisms as well. It is uncertain which model organisms will be targeted with whole genome or focused libraries using CRISPR as the availability of whole-genome sequence information expands.

Issues such as target site accessibility have not been experimentally explored. There are indications that sgRNA targeting is not influenced by local chromatin structure, whereas other reports propose that chromatin accessibility contributes to Cas9 binding (Kuscu et al., 2014; Wu et al., 2014). Some cases report that increased cleavage efficiency correlates with high GC content adjacent to the PAM (Ren et al., 2014). In general, the contribution of sequence and chromatin structure on gene editing needs to be more formally addressed. In addition, understanding the contribution of PAM proximal and PAM distal sequences for Cas9 target binding and cleavage are crucial for the design of high quality libraries. It was suggested that the major determinant for on-target activity is a “seed sequence” proximal to the PAM motif (Kuscu et al., 2014; Wu et al., 2014), whereas the PAM distal sequence is required for efficient target recognition and cleavage, but less so for initial binding (Cencic et al., 2014). This is of particular importance for determining potential off-target activities.

Another key point of consideration is that CRISPR library design to date has focused on gene exon elements. There are multiple other genomic elements that can be targeted, such as long non-coding RNAs, UTR regions or the mitochondrial genome. Further, this may provide a way to dissect the function of introns, an understudied area of functional genomics (Chorev and Carmel, 2012). It can be expected that developments in these areas will flourish in the future. A large variety of sgRNA libraries can be designed, each for specific functionalization or target regions – possibly too many for most screening facilities to host. Thus, it remains to be seen which ones will be commercially viable.

Most current screening efforts focus on use of the *S. pyogenes* Cas9 protein, but it can be expected that Cas9 isoforms from other organisms have advantages in terms of delivery and functionality. The use of other type II Cas proteins can offer an opportunity to multiplex and/or use other PAM motifs for increased variety in target sequence recognition. A recent report has very elegantly demonstrated an expansion of the PAM motif repertoire for *S. pyogenes* Cas9 and suggests that engineering a wide range of Cas9s with altered and improved PAM specificities is possible (Kleinstiver et al., 2015). In addition, Cas9 can be directed to cleave single stranded RNA instead of DNA (O’Connell et al., 2014), a property that can be exploited for transcript-based approaches in gene silencing.

Finally, CRISPR screening has become a possibility in 3D models, tissues and whole organisms (Platt et al., 2014; Chen et al., 2015). The generation of a Cre-dependent Cas9 knockin mouse enables the manipulation of genes in specific tissues, for instance by viral or non-viral delivery of sgRNA to the brain or other tissues. Importantly, this technology for the first time enables complex studies of acute modulation of brain-specific phenotypes, which will be key to develop a more thorough understanding of neuronal diseases. Using tissue-specific expression systems, it is thus possible to target a functionalized protein to any location within a whole organism. This truly is a new age in functional genomics.

## Conflict of Interest Statement

The authors declare that the research was conducted in the absence of any commercial or financial relationships that could be construed as a potential conflict of interest.
